# The Underlying Stroke Etiology: A Comparison of Two Classifications in a Rural Setup

**DOI:** 10.7759/cureus.5157

**Published:** 2019-07-17

**Authors:** Avani R Patel, Amar R Patel, Soaham Desai

**Affiliations:** 1 Internal Medicine, Northern California Kaiser Permanente, Fremont, USA; 2 Neurology, Pramukhswami Medical College, Karamsad, IND

**Keywords:** ischemic stroke, rural healthcare, toast classification, ascod classification, atherothrombosis, cardiac pathology, other causes, dissection, small vessel disease, hypertension

## Abstract

Introduction

This study compares the Trial of Org 10172 in Acute Stroke Treatment (TOAST) and the atherothrombosis, small vessel disease, cardiac pathology, other causes, and dissection (ASCOD) classification performed in a rural hospital setup. Stroke is the second leading cause of death after ischemic heart disease with over 9.5 million new cases of ischemic stroke in 2016. Stroke is a complex disease with numerous contributing factors. India needs a standardized stroke classification system, as without one it becomes difficult to collect data on stroke patients, perform follow-ups, and provide appropriate secondary prevention. A standardized stroke classification system would also help in building a nationwide database in order to note epidemiological trends of ischemic stroke. This would also create greater awareness regarding stroke in rural parts of India where healthcare is difficult to access.

Aims and objectives

Our aim was to review all admitted stroke patients’ data and classify their etiology and mechanism based on the TOAST and ASCOD classification systems. The ASCOD classification has yet to be utilized in the Indian population. The two classifications are then compared in order to gain a better insight into which classification is a better fit for the Indian population. Both are based on the etiology of ischemic stroke but the ASCOD classification differs because it gives suitable secondary prevention measures based on the diseases linked to stroke. ASCOD also gives a proper indication of the patient’s present causative factor (similar to TOAST) and other factors that can possibly lead to further recurrences. This is different from TOAST, which denotes only a single cause for stroke and eliminates the possibility of other involved contributing factors.

Materials and methods

All patients involved in the study were admitted to a rural Indian hospital from January 2014 to July 2016. All the relevant clinical details of each patient were then retrieved from the hospital’s electronic medical record system for the study. We then classified all the patients based on the TOAST and ASCOD classification criteria.

Results

Using the ASCOD classification, we found that 179 (86%) patients out of 209 had either atherothrombosis or small vessel disease. The ASCOD classification also showed substantial evidence that the determined stroke mechanism/etiology is interconnected to multiple causal factors in over 50% of patients. In contrast, the TOAST classification had identified a larger number of ischemic stroke patients as having an etiology of other and undetermined causes as compared to the ASCOD classification.

Conclusion

The ASCOD classification is better to use in patients and helps decide the secondary prevention appropriately.

## Introduction

The World Health Organization defines stroke as rapidly developing clinical signs of focal (and sometimes global) disturbance of cerebral function lasting more than 24 hours or leading to death with no apparent cause other than that of vascular origin [1]. Strokes can be divided into hemorrhagic stroke and ischemic stroke. In our study, we aim to compare two different etiological classifications of ischemic stroke. Ischemic stroke has been defined as an episode of neurological dysfunction that is caused by a localized cerebral, spinal, or retinal infarction [2]. It is associated with risk factors such as hypertension, diabetes mellitus, smoking, dyslipidemia, alcohol consumption, drug abuse, previous stroke, previous transient ischemic attack, migraine history, atrial fibrillation, coronary artery disease, and family history of stroke among first and second-degree relatives [3].

Stroke has been determined to be a major leading cause of disability and the second leading cause of death [4]. In 2016, the Global Burden of Diseases, Injuries, and Risk Factors Study (GBD) established that there were 5.5 million (95% uncertainty interval [UI]: 5.3-5.7) deaths and loss of 116.4 million (95% UI: 111.4-121.4) disability-adjusted life-years (DALYs) due to stroke [5]. GBD also reported that the year 2016 had over 9.5 million new cases of ischemic stroke, of which almost 60% occurred in patients under 70 years of age [6]. Also, there were over 2.7 million deaths attributable to ischemic stroke in the year 2016 [6].

Countries like India are seeing an increasing number of stroke cases and believe that larger academic studies can bring more awareness on a national level. This awareness relates to the criteria by which ischemic stroke is classified. India has a vast population, and many differences exist between different regions of the country in relation to stroke. Different studies use different ways of classification regarding stroke etiology and mechanism. A unified system of assessment and classification of patients of ischemic stroke based on etiology will need to be utilized to compare and contrast stroke cases across the country. Two etiologic classifications that are commonly used are the Trial of Org 10172 in Acute Stroke Treatment (TOAST) and the atherothrombosis, small vessel disease, cardiac pathology, other causes, and dissection (ASCOD) classification [7-8]. The TOAST classification subcategories are large artery atherosclerosis (LAA), small vessel disease (SVO), cardiac embolism (CE), other causes, and undetermined causes [7]. The ASCOD classification subcategories are atherothrombosis (A), small vessel disease (S), cardiac pathology (C), other causes (O), and dissection (D) [8]. The TOAST classification pinpoints a single determinant as the cause of stroke. In contrast, the ASCOD classification lists all possible phenotypes that could potentially cause stroke and grades each subcategory according to the level of evidence available [8]. There is a lack of data regarding risk factors, etiology and the mechanism of stroke in India. Worldwide, studies have demonstrated that not only is stroke a major cause of death in the adult population, but there is still a lack of data regarding its etiology. Our aim for this study was to review ischemic stroke patients and classify their stroke etiology based on the two classification systems mentioned earlier. We utilized two systems in this study in order to gain insight into which system is a better fit for describing stroke etiology across the Indian population.

## Materials and methods

**Figure 1:**
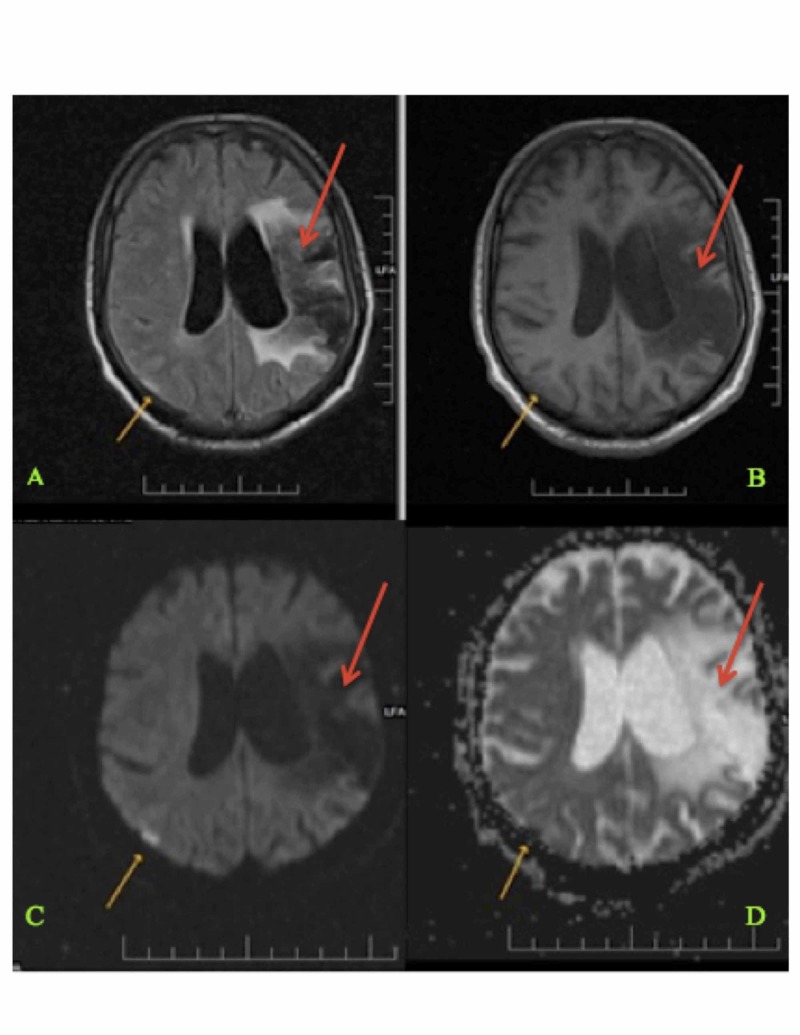
Magnetic Resonance Imaging (MRI) of the Brain Showing Current and Previous Infarction Sites (Panels A-D) Magnetic resonance imaging (MRI) of the brain showing an acute right-sided parietal lobe infarct (shown with yellow arrows) and an old left-sided middle cerebral artery territory infarct (shown with red arrows) in a 63-year-old patient [9].

The study population consisted of ischemic stroke patients admitted to a rural Indian hospital from January 2014 to July 2016. The name of the rural hospital is Shree Krishna Hospital and is located in the western state of Gujarat, India. For the study, 209 patients were selected. The patient’s data were collected from the hospital’s electronic medical record system. Relevant data included each patient’s demographic information, baseline risk factors, presenting complaints, stroke severity, diagnostic evaluations, and secondary prevention treatments. Diagnostic evaluations included brain imaging, computed tomography (CT), magnetic resonance angiography (MRA), electrocardiogram (ECG), and echocardiography (see Figure 1). Secondary prevention consisted of anticoagulant treatment, antiplatelet treatment, statin treatment, and thrombolysis. Each patient’s personal information was removed from the data in order to preserve patient confidentiality. Patients were classified according to the TOAST and ASCOD classification systems [7-8].

The TOAST classification utilizes five subcategories: large artery disease, cardioembolism, small vessel occlusion, other determined etiology, and undetermined etiology. Evidence from the TOAST classification points to a single cause while neglecting other associated diseases. The ASCOD classification includes atherothrombosis, small vessel disease, cardiac pathology, other causes, and dissection. For each patient, ASCOD assigns the probability of each category being responsible for stroke occurrence. We determined the frequency of each stroke etiology/mechanism according to both classification systems and the most predominant mechanism in each classification system.

## Results

**Figure 2:**
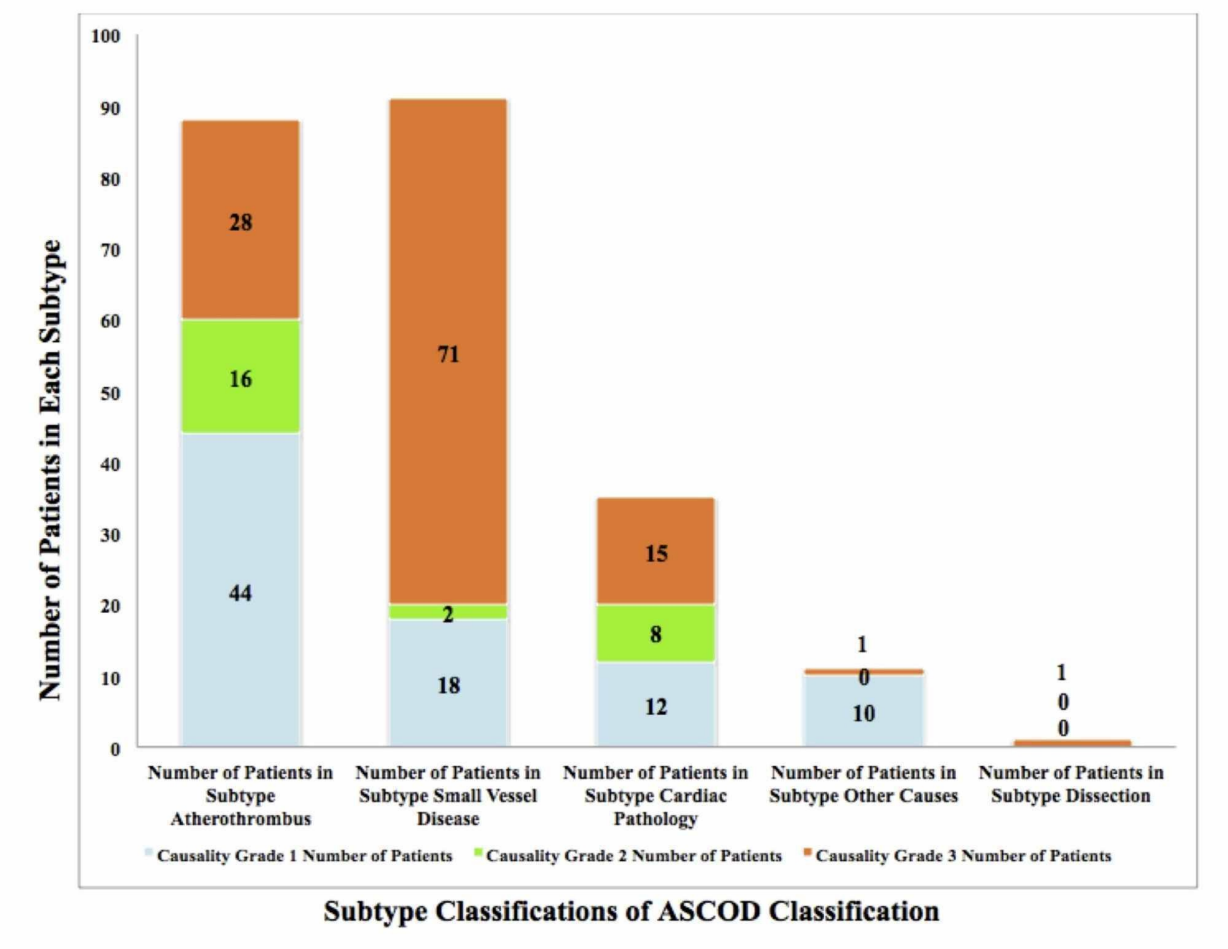
ASCOD Classification of Stroke Patients in the Study A bar graph was created to represent patient distribution into different subtypes of the ASCOD classification (seen with bars). Each subtype was then further classified into different causality grades (blue, green, and orange). With the ASCOD classification, it was determined that A was present in 42% of patients (A1 = 44, A2= 16, A3 = 28), S was present in 44% of patients (S1 = 18, S2 = 2, S3 = 71), C was present in 17% of patients (C1 = 12, C2 = 8, C3 = 15), O was present in 5% of patients (O1= 10), and D was present in 1% of patient. The patient population used comprised 209 people. ASCOD: atherothrombosis (A), small vessel disease (S), cardiac pathology (C), other Causes (O), dissection (D); A1: atherothrombosis grade 1; A2: atherothrombosis grade 2; A3: atherothrombosis grade 3; S1: small vessel disease grade 1; S2: small vessel disease grade 2; S3: small vessel disease grade 3; C1: cardiac pathology grade 1; C2: cardiac pathology grade 2; C3: cardiac pathology grade 3; O1: other causes grade 1.

**Figure 3:**
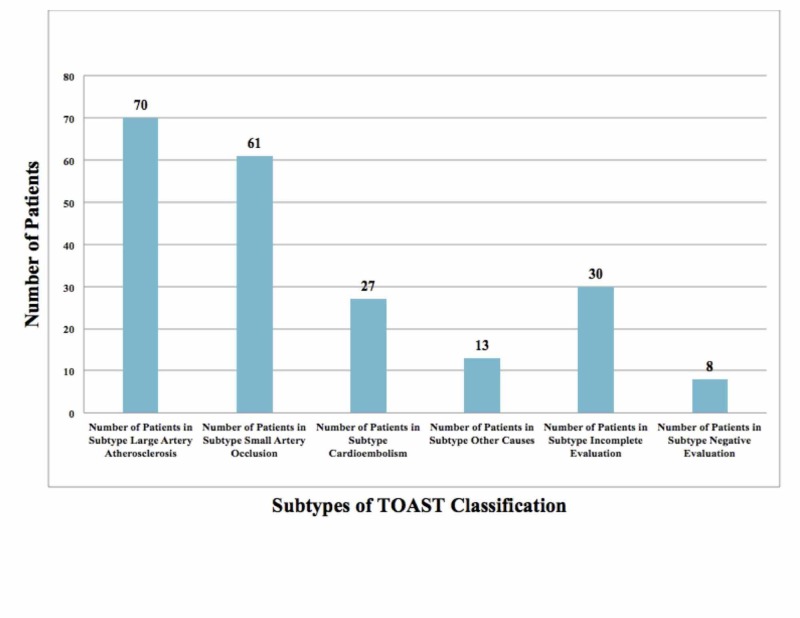
TOAST Classification of Stroke Patients in the Study A bar graph was created to represent patient distribution according to the TOAST classification. The x-axis represents the subtypes of TOAST classification and the y-axis represents the number of patients in each subtype. The study population comprised 209 patients. The TOAST classification showed LAA (70), SVO (61), CE (27), other causes (13), and undetermined (30). TOAST: Trial of Org 10172 in Acute Stroke Treatment; LAA: large artery atherosclerosis; SVO: small vessel occlusion; CE: cardioembolism.

We evaluated a total of 209 patients (mean age: 61 years), of which 64% were men and 36% were women. The baseline characteristics of the study population were identified and evaluated (see Table 1). The prevalence of risk factors in our study was as follows: hypertension (60%), diabetes (32%), ischemic heart disease (11%), heart valve pathology (4%), and dyslipidemia (3%). The determined prevalence of personal habits documented was found to be smoking (14%), tobacco (6%), and alcohol (5%). Despite the recognized association between smoking as a risk factor and stroke incidence, there is a high likelihood that patients underreported their smoking habit as it is considered socially unacceptable in that area of the world [10]. Using the ASCOD classification, we found that A was present in 42% of patients (A1 = 21%, A2 = 8%, A3 = 13%), S was present in 44% of patients (S1 = 9%, S2 = 1%, S3 = 34%), C was present in 17% of patients (C1 = 6%, C2 = 4%, C3 =7 %), O was present in 5% of patients (O1 = 5%), and D was present in <1% of patients (see Figure 2). The TOAST classification showed LAA (33%), SVO (29%), CE (13%), other causes (6%), and undetermined (18%) (see Figure 3). In the ASCOD classification, there was an overlap of disease between grades 1 and 2 (3%) and when extended to grade 3 the overlap was 26%. 

**Table 1:** Baseline Characteristics of the Study Population The prevalence of risk factors in the study population (209 patients) is presented in the above table. Certain factors such as hypertension (60%) and diabetes (32%) were represented more. No: number; CT: computed tomography; MRA: magnetic resonance angiography; ECG: electrocardiogram.

Characteristics Identified	Study Population n = 209 patients, No. (%)
Median age of patients	61
Number of women	75 (36%)
Risk factors	
Hypertension	125 (60%)
Diabetes	66 (32%)
Ischemic heart disease	22 (11%)
Dyslipidemia	7 (3%)
Coronary revascularization	5 (2%)
Heart valve pathology	8 (4%)
Smoking	30 (14%)
Tobacco	13 (6%)
Alcohol	11 (5%)
Diagnostic evaluation	
Brain imaging	175 (84%)
CT or MRA	127 (61%)
ECG	209 (100%)
Echocardiography	107 (51%)
Secondary prevention	
Anticoagulant treatment	18 (9%)
Antiplatelet treatment	158 (76%)
Statin treatment	160 (77%)
Thrombolysis	5 (2%)

Diagnostic evaluation was necessary in both classifications to further categorize each patient. In our study, we found the prevalence of conclusive brain imaging (84%), CT angiography (CTA) or magnetic resonance angiogram (MRA) (61%), ECG (100%), and echocardiography (51%). The prevalence of incomplete evaluations consisting of brain imaging (16%), CTA or MRA (39%), or echocardiography (49%) was indicated by discharges against medical advice. These patients had financial restrictions, they refused to give consent for the procedure, or their power of attorney requested a transfer to another hospital.

## Discussion

The TOAST classification was used to classify subtypes of ischemic stroke. TOAST helped neurologists to further determine the treatment, the prognosis, and the recurrence of stroke in these patients [7]. Similarly, the ASCOD classification is a phenotypic classification that broadly lists all the possible causes that could lead to stroke. Based on the degree of evidence, each disease can be certain, uncertain, unlikely, negative, or incompletely studied as a link to stroke [8]. Both classifications require an extensive workup, and incomplete investigations can lead to deficiencies in proper classification.

The ASCOD classification has yet to be utilized in the current scenario in India. Many studies online still research stroke subtypes by using the TOAST classification. The limitation of the TOAST classification is that it focuses on a single cause for stroke. By directing treatment to a single cause, inadequate treatment is being given to patients. Clinicians can potentially overlook the possibility of other diseases that if left undiagnosed, could lead to stroke recurrence. The main advantage of the ASCOD classification is that it gives a proper picture of the patient’s present causative factor and other factors which can possibly lead to further recurrences. In our study population (n= 209 patients), we found an overlap of 3% of patients (ASCOD grades 1-2) and 55% of patients (ASCOD grades 1-3). This showed substantial evidence that stroke mechanism/etiology is interconnected to multiple causes. In contrast, the TOAST classification denotes only a single cause for stroke but eliminates the possibility of other involved contributing factors. The ASCOD classification gives suitable secondary prevention measures based on the diseases linked to stroke. National application of this classification can lead to better primary and secondary prevention in these patients.

As of now, there are no available studies for the application of the ASCOD classification in an Indian setup. Few studies have utilized the TOAST classification for subtype determination (see Table 2). As physicians, we hope to incorporate more ASCOD classifications in our approach to ischemic stroke, possibly because in the past couple of years more of the world has adopted the ASCOD classification (see Table 3).

**Table 2:** TOAST Classification Studies Conducted in India n: number of patients; LAA: large artery atherosclerosis; SVO: small vessel disease; CE: cardioembolism; TOAST: Trial of Org 10172 in Acute Stroke Treatment.

Location in India	Authors	Age Group	LAA	SVO	CE	Other Causes	Undetermined Causes
Pramukhswami Medical College, Karamsad, Gujarat, India (n = 209)	Current study	2-96 years	33%	29%	13%	6%	18%
Nizam’s Institute of Medical Sciences, Hyderabad, Telangana, India (n = 392) [11]	Kaul et al. 2002	2-97 years	41%	18%	10%	4%	27%
All India Institute of Medical Sciences, Delhi, India (n = 440) [12]	Dash et al. 2014	18-45 years	4.7%	6.8%	14%	17.3%	57%

**Table 3:** TOAST and ASCOD/ASCO Stroke Etiology Classification Studies Conducted Internationally TOAST: Trial of Org 10172 in Acute Stroke Treatment; CE: cardioembolism; LAA: large artery atherosclerosis; SVO: small vessel occlusion; ASCOD: atherothrombosis (A), small vessel disease (S), cardiac pathology (C), other causes (O), and dissection (D).

Author Name and Year of the Study	Study Population	Observation
Gökçal et al. 2017 [13]	151 patients	Using the TOAST classification, patient stroke etiology was classified into undetermined (41.1%), CE (19.2%), LAA (13.2%), SVO (11.3%), and other causes (15.2%). Compared to the TOAST classification, ASCO classification assigned fewer patients to undetermined etiology subtype (26.5%, p<0.001) and SVO category (21.9%, p<0.001). ASCO also assigned more patients to the LAA group (16.6%).
Arsava et al. 2017 [14]	1,816 patients	The classification systems were different in their ability to assign stroke etiologies to known subtypes; the size of the undetermined category was 53% per the TOAST classification and 42% per the ASCO classification (p < 0.001 for all binary comparisons).
Markaki et al. 2013 [15]	101 patients, 84 with ischemic stroke and 17 with a TIA	There was a moderately high agreement between the TOAST and ASCO classifications in all subtypes. Along with the classification, the one- and four-year mortality rates were observed during a mean observation period of 28 months, during which 26 patients died. The one- and four-year mortality rates, respectively, were 0% and 4% in LAA, 23% and 36% in CE, 0% in SVO, 63% and 100% in unknown etiology, and 12% and 29% in the cryptogenic subtype. For the ASCO classification, the one-year and four-year mortality rates, respectively, were 0% and 6% in LAA, 25% and 36% in CE, 0% in SVO, 0% and 14% in LAA + CE, 16% and 36% in SVO + CE, and 56% and 100% in the undetermined etiology despite complete workup.
Shang et al. 2012 [16]	425 patients with first time ischemic stroke	There was a moderately high agreement between the TOAST and ASCO classification in all subtypes except the “undetermined” etiology subtype (16.2% vs. 15.5 %, p = 0.795).
Wolf et al. 2012 [17]	103 patients	There was a high agreement between the ASCO and TOAST classifications. With ASCO, grades 1-3 were identified in 60.19% A, 75.73% S, 49.51% C, and 3.88% O. Around 68.93% of the patients were classified in more than one category, and only 3.88% remained completely undetermined. With the TOAST classification, the distribution was 9.71% in A, 23.30% in S, 34.95% in C, 1.94% in O, and 30.10% in the undetermined subtype.

The Hyderabad study showed a similar median age of patients at 54 years compared to a median age of 61 years in our study [11]. The predominant subtype of ischemic stroke was LAA (41-33%). Undetermined etiology was the second most common in that study [11]. This was mainly attributed to the lack of the new algorithm proposed by the Stop-Stroke Study TOAST in 2005 [18]. The new modifications to the TOAST classification expanded the definitions of SVO and LAA that then decreased the undetermined subtype to 4% [18]. Our study still had a large proportion of undetermined cases (18%), which were due to incomplete evaluation (14%) and negative evaluation (4%). The reasons for incomplete evaluation included financial restraints of the patients, negative consent by the relatives, request for transfer, or death of the patient. In India, financial restraints proved to be the greatest barrier to proper evaluation of a stroke patient. For additional information on the TOAST and ASCOD classifications, see the appendix for Tables 4-11.

## Conclusions

Stroke is a complex disease with numerous contributing factors. Without a standardized protocol, it becomes difficult to collect data on patients, follow up, and provide treatment. The ASCOD classification is a better fit for patients of the Indian population and helps in deciding secondary prevention appropriately. However, we need to continue evaluating its applicability by motivating more physicians to generate larger prospective studies utilizing the ASCOD classification. Only with further studies can physicians come closer to a more standardized approach to ischemic stroke classification.

## Appendices

**Table 4:** TOAST Classification [7] A table was created representing the different subtypes of the TOAST Classification [7]. TOAST: Trial of Org 10172 in Acute Stroke Treatment.

Trial of Org 10172 in Acute Stroke Treatment (TOAST) Classification [7]						
Classification of Subtypes of Ischemic Stroke						
Large Artery Atherosclerosis	Small Vessel Occlusion	Cardioembolism	Stroke of Other Determined Etiology	Stroke of Undetermined Etiology	Stroke of Undetermined Etiology with a Negative Evaluation	Stroke of Undetermined Etiology with an Incomplete Evaluation

**Table 5:** ASCOD Classification [8] A table was created representing the different causality grades, as an underlying etiology of ischemic stroke as per a subcategory of the ASCOD classification [8]. ASCOD: atherothrombosis, small vessel disease, cardiac pathology, other causes, and dissection classification.

ASCOD Classification [8]				
Classification of Subtypes of Ischemic Stroke				
Atherothrombosis (A)	Small Vessel Disease (S)	Cardiac Pathology (C)	Other Causes (O)	Dissection (D)

**Table 6:** Atherothrombus (A) ASCOD Classification with Causality Grades [8] A table was created representing the different causality grades, as an underlying etiology of ischemic stroke as per a subcategory of the ASCOD classification [8]. A: atherothrombosis; MR-DWI: magnetic resonance diffusion-weighted imaging; US-Duplex: ultrasound duplex; CTA: computed tomography angiography; MRA: magnetic resonance angiography; XRA: X-ray angiography; US-TCD: ultrasound transcranial Doppler; TEE: transesophageal echocardiography.

The Causality Grades [8]	ASCOD Atherothrombosis (A) Phenotypes According to Classification [8]						
A1: potentially causal. A stroke that is atherothrombotic is defined as one of the following:	An ipsilateral atherosclerotic stenosis of 50-99% in an intracranial or extracranial artery supplying the ischemic field.	An ipsilateral atherosclerotic stenosis <50% in an intracranial or extracranial artery with an endoluminal thrombus supplying the ischemic field.		A mobile thrombus in the aortic arch.		An ipsilateral arterial occlusion in an intracranial or extracranial artery with evidence of underlying atherosclerotic plaque supplying the ischemic field.	
A2: the causal link is uncertain. Defined as potentially one of the following:	An ipsilateral atherosclerotic stenosis of 30-50% in an intracranial or extracranial artery supplying the ischemic field.	An aortic plaque ≥ 4 mm without a mobile lesion.					
A3: the causal link is unlikely, but the disease is present. One or more of the following may be seen:	A plaque (stenosis <30%) in an intracranial or extracranial artery, which is ipsilateral to the infarct area.	An aortic plaque <4 mm without a mobile thrombus.	A stenosis of any degree that is not supplying the infarct area.		A present history of myocardial infarction, coronary revascularization, or peripheral arterial disease.		An ipsilateral or bilateral atherosclerotic stenosis of 50–99% with bihemispheric MR-DWI lesion present.
A0: atherosclerosis is not detected. In order to rule out atherosclerosis, the following should be looked for:	A negative finding for an extracranial arterial stenosis on US-duplex, CTA, MRA, XRA, or autopsy.	A negative finding for an intracranial arterial stenosis on US-TCD, CTA, MRA, XRA, or autopsy.	A negative finding for an aortic arch atheroma: TEE or CTA with specific assessment of the aortic arch.				
A9: an incomplete workup done on the patient. There will be a lack of tests performed such as the following:	US-duplex, US-TCD or CTA, or MRA, or XRA or autopsy has not been performed.	The minimum workup is extra- and intracranial assessment of the cerebral arteries.	The maximum workup also includes TEE and CTA of the aortic arch.				

**Table 7:** Small Vessel Disease (S) ASCOD Classification with Causality Grades [8] A table was created representing the different causality grades, as an underlying etiology of ischemic stroke as per a subcategory of the ASCOD classification [8]. ASCOD: atherothrombosis, small vessel disease, cardiac pathology, other causes, and dissection classification; MRI-DWI: magnetic resonance diffusion-weighted imaging; MRI: magnetic resonance imaging; CT: computed tomography; FLAIR: fluid-attenuated inversion recovery; GRE: gradient echo imaging; DWI: diffusion-weighted imaging.

The Causality Grades [8]	ASCOD Small Vessel Disease (S) Phenotypes According to Classification [8]			
S1: potentially causal. A stroke that will have a combination of a lacunar artery infarction on an MRI-DWI in an area corresponding to the symptoms and at least one out of the three following criteria:	One or several small deep older infarct(s) of lacunar type in other territories.	The patient has severe white matter lesions, small vessel ischemia, microbleeds, or severe dilatation of perivascular spaces.		
S2: the causal link is uncertain. It is defined as potentially one of the following:	Only one recent lacunar infarction and no other abnormality is seen on MRI or CT.	The patient has a clinical syndrome suggestive of a deep branch artery stroke, without an ischemic lesion in the appropriate area.		
S3: the causal link is unlikely, but the disease is present.	There are severe white matter lesions representative of small vessel ischemia visible on an MRI or CT scan.	There are microbleeds seen on a T2-weighted MRI.	There is a severe dilatation of the perivascular space visible on T2-weighted MRI.	There are one or several old, small, and deep infarcts of the lacunar type.
S0: small vessel disease is not detected. In order to rule out small vessel disease, look for the following:	Patient has anegative MRI (T2, FLAIR, GRE, DWI).	There is no appropriate clinical syndrome suggestive of a deep branch artery stroke.		
S9: an incomplete workup done on the patient. There will be a lack of tests performed such as:	MRI	CT scan		

**Table 8:** Cardiac Pathology (C) of the ASCOD Classification with Causality Grades [8] A table was created representing the different causality grades, as an underlying etiology of ischemic stroke as per a subcategory of the ASCOD classification [8]. ASCOD: atherothrombosis, small vessel disease, cardiac pathology, other causes, and dissection classification; PFO: patent foramen ovale; DVT: deep venous thrombosis; ASA: atrial septal aneurysm; ECG: electrocardiogram; TEE: transesophageal echocardiography; TTE: transthoracic echocardiography; CT: computed tomography; MRI: magnetic resonance imaging.

The Causality Grades [8]	ASCOD Cardiac Pathology (C) Disease Phenotypes According to Classification [8]							
C1: potentially causal. A stroke that is cardiogenic is defined as an ischemic lesion and has signs of systemic embolism with detection of at least one of the following causes:	Mitral stenosis, mechanical valve, myocardial infarction within four weeks, preceding the cerebral infarction, mural thrombus in the left cavities, aneurysm of the left ventricle, a history or presence of documented atrial fibrillation (either paroxysmal, persistent or permanent) or atrial flutter with or without left atrial thrombus or spontaneous echo, atrial disease (tachycardia-bradycardia syndrome), dilated or hypertrophic cardiomyopathies, left ventricle ejection fraction <35%, endocarditis, intracardiac mass, PFO and thrombus in situ, PFO and concomitant pulmonary embolism or proximal DVT preceding the index cerebral infarction, aforementioned cardiac pathologies (C1) with single or without obvious cerebral ischemic lesion							
C2: the causal link is uncertain. Regardless of the cardiogenic stroke pattern, there may be:	PFO + atrial septal aneurysm.	PFO and pulmonary embolism or proximal DVT concomitant but not preceding the index cerebral infarction.		An intracardiac spontaneous echo contrast.		Apical akinesia of the left ventricle and decreased ejection fraction (but >35%) in the patient.	A history of myocardial infarction or palpitation, and multiple brain infarction, repeated either bilaterally or in two different arterial territories.	No direct cardiac source identified but multiple brain infarctions present and/or evidence of systemic emboli.
C3: the causal link is unlikely, but the disease is present. It is defined as potentially one of the following:	PFO, ASA, strands, mitral annulus calcification, calcification aortic valve, non-apical akinesia of the left ventricle, transient atrial fibrillation less than 1 minute in duration, atrial hyperexcitability.							
C0: cardiac pathology is neither detected nor suspected. Workup needed to rule out cardiac pathology:	The minimum workup needed is a negative ECG and an examination by a cardiologist.				The maximum workup needed is a negative ECG/telemetry/24-hour Holter ECG/long-term ECG recording, a negative TEE, a negative TTE for PFO and assessment of left ventricle, a negative cardiac CT/MRI, and a negative abdominal CT/MRI.			
C9: an incomplete workup done on the patient. Minimum workup needed to rule out cardiogenic shock:	ECG		An examination by a trained cardiologist in the absence of cardiac imaging.					

**Table 9:** Other Causes (O) ASCOD Classification with Causality Grades [8] A table was created representing the different causality grades as an underlying etiology of ischemic stroke as per a subcategory of the ASCOD classification [8]. ASCOD: atherothrombosis, small vessel disease, cardiac pathology, other causes, and dissection classification; GPL: IgG phospholipid units; DVT: deep venous thrombosis; PE: pulmonary embolism; ESR: erythrocyte sedimentation rate; CRP: C-Reactive protein.

The Causality Grades [8]	ASCOD Other Causes (O) Phenotypes According to Classification [8]			
O1: potentially causal. A stroke that is a result of other causes is defined as one of the following:	Dolichoectasia, or elongated, distended, and tortuous cerebral arteries that may present with compressive or ischemic symptoms.	A ruptured intracranial aneurysm with or without vasospasm of the artery supplying the infarcted area.	Polycythemia vera or thrombocythemia, systemic lupus, disseminated intravascular coagulation, antiphospholipid antibody syndrome, Fabry disease, coexisting meningitis, sickle cell disease, severe hyperhomocysteinemia, Horton disease, other cerebral inflammatory or infectious angiitis, Moyamoya disease, etc.	
O2: the causal link is uncertain. Regardless of the stroke pattern, there may be:	A saccular aneurysm with suspicion of embolism.			A coincidental migraine attack with lasting neurological deficit.
O3: the causal link is unlikely, but the disease is present. It is defined as potentially one of the following:	Arteriovenous malformation, thrombocytosis, antiphospholipid antibody <100 GPL units, homocysteinemia <40 μmol/L, malignoma with an associated hypercoagulation, DVT or PE, and/or recent chemotherapy.			
O0: it is neither detected nor suspected. In order to rule out other causes, there needs to be negative:	Cerebrospinal fluid, complete hemostasis, cerebral arterial imaging, family history of inherited disease, ESR, CRP, platelet count, leukocytes, and eosinophilic counts, hematocrit, and specific tests according to the suspected disease such as a genetic test or retinal angiography for Susac syndrome.			
O9: an incomplete workup done on the patient.	It is not possible to completely exclude other causes based on available diagnostic tests and stroke-specific history.			

**Table 10:** Dissection (D) ASCOD Classification with Causality Grades [8] A table was created representing the different causality grades as an underlying etiology of ischemic stroke as per a subcategory of the ASCOD classification [8]. ASCOD: atherothrombosis, small vessel disease, cardiac pathology, other causes, and dissection classification; MRI: magnetic resonance imaging; TOF-MRA: time-of-flight magnetic resonance angiography; CT: computed tomography; XRA: X-ray angiography; CTA: computed tomography angiography; MRA: magnetic resonance angiography; US: ultrasound.

The Causality Grades [8]	ASCOD Dissection (D) Disease Phenotypes According to Classification [8]				
D1: potentially causal. A stroke caused by dissection can be a result of one of the following:	An arterial dissection demonstrated by a hypersignal on FAT-saturated MRI, autopsy, TOF-MRA, CT scans, increased arterial diameter.			A demonstration of an arterial dissection by an indirect demonstration or by less sensitive or less specific diagnostic test (XRA, echocardiography, CTA, MRA, US) like an arterial stenosis seen without demonstration of the arterial wall hematoma	
D2: the causal link is uncertain. It is defined as potentially one of the following:	An arterial dissection diagnosed based on a suggestive clinical history like a past history of dissection or Horner’s syndrome.			If there is imaging evidence of fibromuscular dysplasia of a cerebral artery of an involved cerebral field present.	
D3: the causal link is unlikely, but the disease is present. Defined as potentially one of the following;	There is kinking or dolichoectasia (elongated, distended, and tortuous cerebral arteries that may present with compressive or ischemic symptoms) without complicated aneurysm or plicature.			There are arteries not implicated in the current ischemia that have evidence of fibromuscular dysplasia.	
D0: dissection is neither detected nor suspected. In order to rule it out, the following should be done:	A negative fat-saturated MRI.	A normal XRA.	There is a lack of clinical suspicion of dissection.	There are negative extra- and intracranial cerebral artery evaluations.	A negative cardiac evaluation.
D9: an incomplete workup done on the patient. Minimum workup needed to rule out dissection is:	In patients younger than 60 years and with no evidence of A1, A2, S1, C1, or O1, should undergo MRI or XRA within 15 days of symptom onset.				

**Table 11:** Classification System, Types, Evidence Grade, Advantages, and Disadvantages [7-8] TOAST: Trial of Org 10172 in Acute Stroke Treatment; ASCOD: atherothrombosis, small vessel disease, cardiac pathology, other causes, and dissection classification.

Classification Systems	Type of Classification	Evidence Grade	Advantages	Disadvantages
TOAST Classification [7]	Causative	Probable/possible	Convenient; can be used in clinical practice	Usage depends on the clinical expertise of the physician; overestimates the undetermined group; evidence grades are neglected
ASCOD Classification [8]	Phenotypic	Disease absent/potentially causal/causal link/uncertain causal link/unlikely because of insufficient workup	Comprehensive workup; minimal and maximal workup defined; overview of all pathologies causing stroke; correlates well with clinical studies; good validity	A complicated classification; difficult to use in clinical practice

## References

[1] Aho K, Harmsen P, Hatano S, Marquardsen J, Smirnov VE, Strasser T (1980). Cerebrovascular disease in the community: results of a WHO collaborative study. Bull World Health Organ.

[2] Sacco RL, Kasner SE, Broderick JP (2013). An updated definition of stroke for the 21st century: a statement for healthcare professionals from the American Heart Association/American Stroke Association. Stroke.

[3] Das SK, Banerjee TK, Biswas A (2007). A prospective community-based study of stroke in Kolkata, India. Stroke.

[4] Katan M, Luft A (2018). Global burden of stroke. Semin Neurol.

[5] GBD 2016 Stroke Collaborators (2019). Global, regional, and national burden of stroke, 1990-2016: a systematic analysis for the Global Burden of Disease Study 2016. Lancet Neurol.

[6] (2019). World Stroke Organization global stroke fact sheet. https://www.world-stroke.org/images/WSO_Global_Stroke_Fact_Sheet_final.pdf.

[7] Adams HP Jr, Bendixen BH, Kappelle LJ, Biller J, Love BB, Gordon DL, Marsh EE 3rd (1993). Classification of subtype of acute ischemic stroke. Definitions for use in a multicenter clinical trial. TOAST. Trial of Org 10172 in Acute Stroke Treatment. Stroke.

[8] Amarenco P, Bogousslavsky J, Caplan LR, Donnan GA, Wolf ME, Hennerici MG (2013). The ASCOD phenotyping of ischemic stroke (updated ASCO phenotyping). Cerebrovasc Dis.

[9] Patel A R, Patel A R, Desai S (2019). Acute hemiballismus as the presenting feature of parietal lobe infarction. Cureus.

[10] Boehme AK, Esenwa C, Elkind MS (2017). Stroke risk factors, genetics, and prevention. Circ Res.

[11] Kaul S, Sunitha P, Suvarna A, Meena AK, Uma M, Reddy JM (2002). Subtypes of ischemic stroke in a metropolitan city of south India (one year data from hospital based stroke registry). Neurol India.

[12] Dash D, Bhashin A, Pandit AK, Tripathi M, Bhatia R, Prasad K, Padma MV (2014). Risk factors and etiologies of ischemic strokes in young patients: a tertiary hospital study in north India. J Stroke.

[13] Gökçal E, Niftaliyev E, Asil T (2017). Etiological classification of ischemic stroke in young patients: a comparative study of TOAST, CCS, and ASCO. Acta Neurol Belg.

[14] Arsava EM, Helenius J, Avery R (2017). Assessment of the predictive validity of etiologic stroke classification. JAMA Neurol.

[15] Markaki I, Franzén I, Talani C, Loizou L, Kostulas N (2013). Long-term survival of ischemic cerebrovascular disease in the acute inflammatory stroke study, a hospital-based cohort described by TOAST and ASCO. Cerebrovasc Dis.

[16] Shang Wy, Liu Jy (2012). Stroke subtype classification: a comparative study of ASCO and modified TOAST. J Neurol Sci.

[17] Wolf ME, Sauer T, Alonso A, Hennerici MG (2012). Comparison of the new ASCO classification with the TOAST classification in a population with acute ischemic stroke. J Neurol.

[18] Ay H, Furie KL, Singhal A, Smith WS, Sorensen AG, Koroshetz WJ (2005). An evidence-based causative classification system for acute ischemic stroke. Ann Neurol.

